# Accessibility of COVID-19 Vaccination Centers in Germany via Different Means of Transport

**DOI:** 10.1007/s42489-021-00088-x

**Published:** 2022-01-17

**Authors:** Stefan Neumeier

**Affiliations:** Thünen-Institut für Ländliche Räume, Bundesallee 64, 38116 Braunschweig, Germany

**Keywords:** Thünen-Accessibility model, Raster-based accessibility analysis, COVID-19, SARS-CoV-2, Vaccination, Germany

## Abstract

In late 2020, as soon as the approval of the first vaccines against the severe acute respiratory syndrome coronavirus 2 (SARS-CoV-2) became foreseeable in line with the normative political goal of providing comparable living conditions to all residents of Germany irrespective of where they live, the German national government’s national vaccination strategy called for the widespread establishment of COVID-19 vaccination centers. As the vaccination program has been rolled out, difficulties in accessing vaccination centers have been reported. Against this background, the paper considers the questions whether, where and for whom spatial inequalities in COVID-19 vaccination center accessibility in Germany might exist. Such an understanding might help to prepare for future situations when adequate disaster response requires, similar to the COVID-19 pandemic, the government to quickly reach great parts of the population in an efficient manner. To approach this question, we examine the accessibility by the means of transport foot, bicycle, car and public transport at small scale based on an accessibility model from the point of view of the “households”. We found that in contrast to the common belief COVID-19 vaccination center accessibility or inaccessibility in Germany does not seem to be a spatial phenomenon cheating non-rural regions and discriminating rural regions as anticipated, it is instead strongly dependent on people’s individual mobility capabilities in both rural and urban areas.

## Abstract (de)

Als Ende 2020 absehbar war, dass eine Zulassung der ersten Impfstoffe gegen das Severe Acute Respiratory Syndrome Corona Virus 2 (SARS-CoV-2) kurz bevorsteht, entschied sich die Bundesregierung zusammen mit den Länderregierungen im Rahmen einer nationalen Impfstrategie für die flächendeckende Etablierung von Impfzentren. Mit Inbetriebnahme der Impfzentren kam vermehrt Kritik an deren Erreichbarkeit auf. Vor diesem Hintergrund geht der Artikel der Frage nach ob im Hinblick auf die Impfzentrenerreichbarkeit in Deutschland räumliche Ungleichheiten existieren und falls ja, wo und für wen. Wichtig sind solche Erkenntnisse v. a. für die Vorbereitung auf zukünftige Katastrophen-Situationen bei denen eine adäquate Katastrophenhilfe - ähnlich der Reaktion auf die COVID-19 Pandemie - erfordert schnell und effizient große Teile der Bevölkerung zu versorgen. Um diese Fragen zu beantworten, haben wir mit Hilfe eines Erreichbarkeitsmodells kleinräumig und flächendeckend für Deutschland die Erreichbarkeit der Impfzentren mit den Verkehrsträgern Pkw, Fahrrad, Fuß und öffentlicher Personenverkehr aus Sicht der Haushalte analysiert. Als Ergebnis der Analyse lässt sich festhalten, dass entgegen der landläufigen Wahrnehmung bei der Erreichbarkeit der COVID-19 Impfzentren keine gravierenden räumlichen Ungleichheiten existieren, so dass z.B. ländliche Räume generell schlechter gestellt wären als urbane. Erreichbarkeitsunterschiede sind vielmehr auf Unterschiede in den individuellen Mobilitätsmöglichkeiten der Bürger zurückzuführen.

## Introduction

The first cases of a new disease caused by a so-far unknown coronavirus, later known under the name “Severe Acute Respiratory Syndrome Coronavirus 2” (SARS-CoV-2), were reported In Wuhan, China, in early December 2019 (World Health Organization 2021). As with other already known coronaviruses, like the Severe Acute Respiratory Syndrome (SARS) (*R*_0_: 2·0–3·0) and the Middle East Respiratory Syndrome (MERS) (*R*_0_: 0·9), SARS-CoV-2 (*R*_0_: 1·8–3·6), COVID-19 proved to be transmissible from human to human but with a much greater infectivity and a mortality of 0·6–2·8% in people younger than 65 years out of all deaths (Petersen et al. 2020; World Health Organization 2021). Until January 2020, the reported cases and deaths by COVID-19 already exceeded those of SARS or MERS and later caused an escalating number of reported infections in humans as well in China and in other countries around the world (Poon and Peiris 2020; Zowalaty and Järhult 2020). Because of its infectivity, mortality and world-wide spread, on January 30, 2020, the World Health Organization put COVID-19 in the list of Public Health Emergencies of International Concern (PHEIC) (Al-Qahtani 2020; Poon and Peiris 2020). In Germany, the first reported case of SARS-CoV-2 occurred in Bavaria on January 27, 2020. (Bundesgesundheitsministerium n. d.). Until the end of February 2020, further cases were reported in Bavaria, North Rhine-Westphalia and Baden-Württemberg. However, the situation still seemed to be controllable (Bundesgesundheitsministerium n. d.). However, by March 10, 2021, all federal states reported increasing cases of infections with SARS-CoV-2. As a reaction to the ongoing worldwide spread of SARS-CoV-2 in combination with increasing cases of severe illnesses and death, on March 11th, the World Health Organization (WHO) declared SARS-CoV-2 a pandemic (Quarks 2020). In Germany, on March 28, 2020, the federal government followed the WHO and declared the SARS-CoV-2 outbreak an epidemic situation of national concern.^1^ As it became apparent that the SARS-CoV-2 pandemic will only be controllable if great parts of the population develop an immunity against the SARS-CoV-2 virus (Wein 2021), virologists worldwide began to seek and develop vaccines effective against SARS-CoV-2, hoping to be able to control the virus outbreak with the help of a targeted vaccination strategy.

In Germany, in late 2020, as soon as the approval of the first vaccines against SARS-CoV-2 became foreseeable, the German Federation and the federal states passed a national vaccination strategy in preparation of the upcoming inoculation campaign (Bundesgesundheitsministerium 2020).

As was the case in many countries, Germany’s national vaccination strategy involved delivering the vaccines first to the most vulnerable members of the population (e.g., elderly in nursing homes) (Robert Koch Institut 2020a) Intriguingly, due to the country’s strong federalism and the paramount political goal of making sure that no one is disadvantaged by where they live, the strategy also involved establishing vaccination centers by the federal states and reaching into the more remote rural areas. Besides, the aim to establish the vaccination center locations in such a manner that access for the whole German population is provided another requirement guiding the establishment of the vaccination centers was the ability to be able to maintain the cold chain for RNA vaccines during the delivery process. According to a study by the Federal Institute for Research on Building, Urban Affairs and Spatial Development (2021) concentrating on the spatial distribution of the population weighed COVID-19 cases rural areas are affected by the pandemic equally to urban agglomerations. However, news coverage reported difficulties in accessing the vaccination centers for parts of the German population^2^ as a result of the establishment of the centers mainly in the urban centers of the rural hinterland. Furthermore, in rural areas as well as in urban agglomerations, some centers are said to be difficult to access, either due to remote locations or due to suboptimal transport connections. Against this background, the paper considers the questions whether, where and for whom spatial inequalities in COVID-19 vaccination center accessibility in Germany might exist, as such an understanding might help in preparation of future situations, where adequate disaster response requires similar to the COVID-19 pandemic to reach great parts of the population in an efficient manner. To answer these questions, we analyze the accessibility of the COVID-19 vaccination centers by the means of transport foot, bicycle, car and public transport from the point of view of the “households” on a small scale based on a 250 m × 250 m analysis grid with the help of an accessibility model.

Four sections follow the introduction. In the next section, we introduce the concept accessibility. In Section Three, data and methodological issues are discussed. This is followed by a presentation and discussion of our findings on COVID-19 vaccination center accessibility in Germany. The final section summarizes the findings and provides conclusions and considerations for future research.

## The Concept Accessibility

Commonly accessibility is defined as expenses caused by gaining access to private or public services measured in travel time, distance or transport costs (Hansen 1959; Schürmann et al. 1997; Schwarze 2005; Bleisch et al. 2003; Dahlgren 2008; Hanson 2009; Vulevic 2016; Great Britain Department for Transport 2012; Albacete et al. 2017). Important aspects to determine accessibility are therefore:

- the origin of an activity (e.g., place of residence)
- the destination(s)—that is the intended activities together with their locations—and
- the effort measured in time, distance or other costs necessary to come from the origin of an activity to the destinations.

The latter is again subject to natural realities, the available itineraries like roads, rails, rivers etc. and the available means of transport used. If the effort to reach the destinations from the origin of an activity is low, one can speak of a good accessibility. If it is high is high, one can speak of a bad accessibility. Nevertheless, the judgment what qualifies as a good or bad accessibility is likely to be rather an individual perception based on experiences, habits, capabilities, etc., than a universally definable threshold. Furthermore, it can be assumed that for different infrastructures different accepted travel times might exist. However, for Germany, for the accessibility of basic services infrastructures provided that no service-specific nominal defined travel times exist, a travel time of up to 15 min can be taken as a rough threshold to differentiate good accessibility from a poor accessibility (Amt für Raumentwicklung und Geoinformation, Kanton St. Gallen 2008; Bundesministerium für Verkehr, Bau und Stadtentwicklung 2011). As to COVID-19 vaccination centers, it can be assumed that the threshold is a little bit higher. The different methods to acquire accessibility indicators can roughly be divided into the following two categories (Bleisch et al. 2003; Schürmann et al. 1997; Schwarze 2005):

- Simple supply indicators that concentrate on aspects like the length of the road network or the number of infrastructure locations per community or
- More complex generic indicators that concentrate on aspects like travel times or length of travel in traffic networks or space.

Because of the relative ease to acquire supply indicators, they are often used by policy and the media. However, they do not allow conclusions on intraregional differences in service provision, neglect the network character of traffic infrastructures as well as the connection between regions, or the fact that not transport facilities but infrastructures that can be reached using transport facilities are the destinations (Spiekermann and Wegener 2008). Such supply indicators deliver only a rough estimation of accessibility situations, neglecting important spatial patterns influencing the accessibility. Furthermore, the deviation from reality of such indicators grows with the size of the base region, an effect known as modifiable areal unit problem (MAUP) (Madelinet al. 2009).

The more complex generic accessibility indicators can again be divided into the following three sub-categories:

- Approaches common in transport sciences based on a prognosis of the traffic situation like gravity models,^3^ opportunity models^4^ and utility models^5^ (Handy and Niemeier 1997; Bleisch 2005; Schulz and Bröcker 2007),
- Approaches common mainly in the regional economy based on spatial interaction models that estimate flows between locations that enable to evaluate the demand for transport services like gravity models,^6^ potential models^7^ and retail models^8^ (Bleisch 2005; Schulz and Bröcker 2007; Rodrigue 2020),
- Approaches focusing on the geographic accessibility like isochrones indicating the number or proportion of destinations reachable within a given travel distance or time, Euclidean distance or distance or travel times within traffic networks(Geurs and Ritsema van Eck 2001; Geurs and van Wee 2004; Hemetsberger and Ortner 2008).

To analyze the accessibility situation of COVID-19 vaccination centers as available to the population, it was decided to concentrate on geographic accessibility by the means of transport car, bicycle, foot and public transport measured in travel time in the respective traffic networks.

## Data and Methodology

**Fig. 1:**
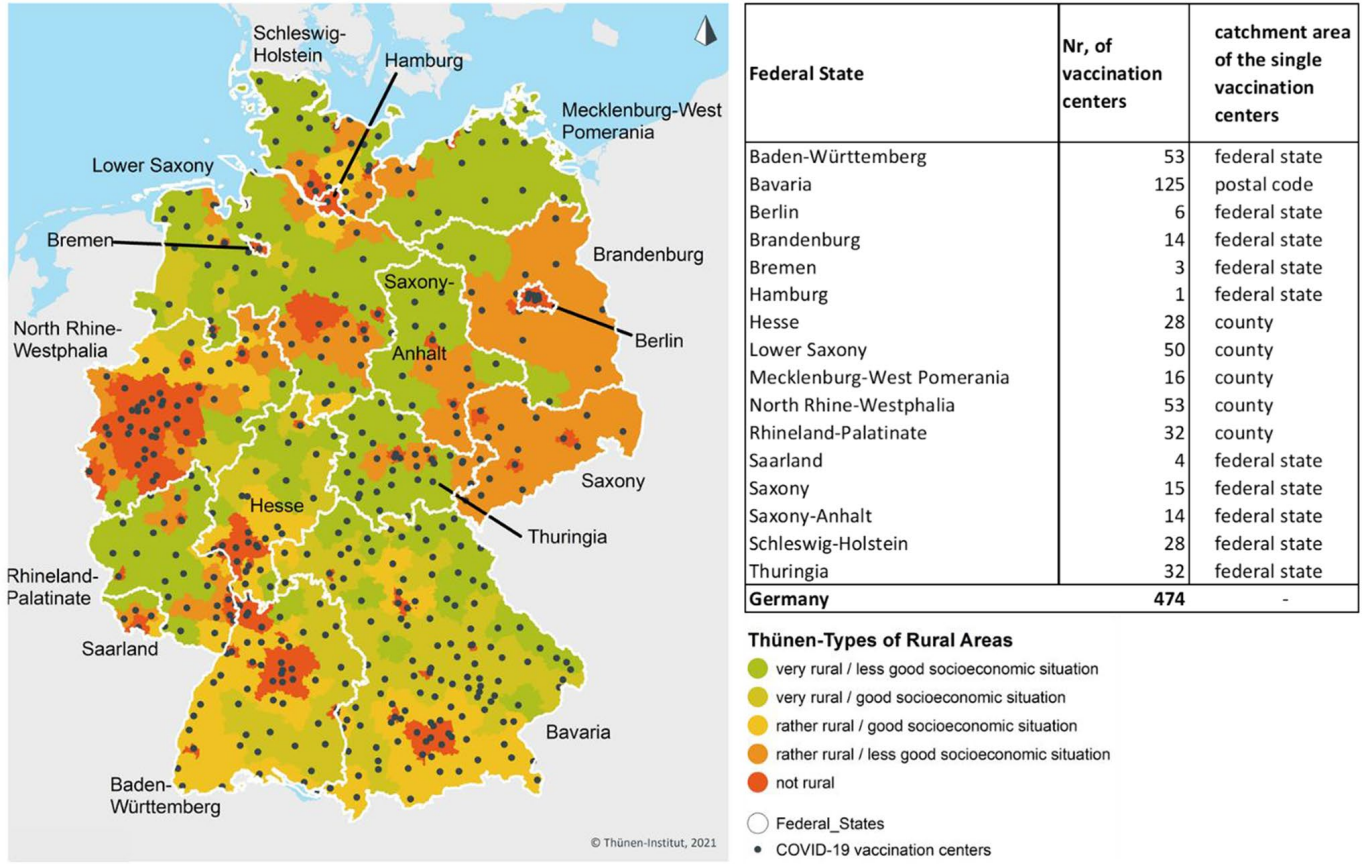
Federal States, Thünen-Types of Rural Areas, locations and number of COVID-19 vaccination centers and catchment area allocation as of end of May 2021. Administrative boundaries: German Federal Office of Cartography and Geodesy (2020); Thünen-Types of Rural Areas: www.landatlas.de/Küpper2016; COVID-19 vaccination centers: OSM, vaccination center location lists of the federal states

The empirical analysis draws on data from following different sources:

- Locational data of the of COVID-19 vaccination centers in Germany based on the COVID-19 vaccination center points of interest contained in the Open Street Map (OSM) as of May 20, 2021 complemented by geocoded address data from official listings of COVID-19 vaccination center locations in all 16 federal states of Germany as of end of May 2021 (see Fig. 1).
- A 250 m × 250 m analysis grid consisting of 57,138,239 grid cells, enriched with population information,^9^ the region types of the Thünen-Typology of rural areas (see Fig. 1)^10^ as well as state, community and county affiliation for every grid cell covering the area of Germany.
- The accessibility analysis by car, bicycle and foot is based on the traffic-network of the OSM.
- The accessibility analysis by public transport is based on the traffic-network of the OSM as well as the public transport time table and route information for the whole German public transport system (long distance traffic, regional transport, rapid transit) stored in the so-called General Transit Feed specification (GTFS) format licensed under Creative Commons 4.0 and provided by gtfs.de at June 3, 2021. As reference time for the public transport analysis, Tuesday, June 8, 2021, 9 a.m. was chosen.

### COVID-19 Vaccination Center Locations

Except for the federal states Bavaria, Saarland and Rhineland Palatinate, the locational data of the COVID-19 vaccination centers are based on the vaccination center locations recorded as points of interest in the OSM as of May 20, 2021. The locational data extracted from the OSM have been aligned with the official vaccination center address lists of the single states^11^ as of end of May 2021 (see Table 1), thereby missing locations in the OSM-extract were amended and wrong entries have been corrected or removed.

As the location data of COVID-19 vaccination centers for the federal states Bavaria, Saarland and Rhineland Palatinate proved to be missing or unreliable, in the OSM extract, locational data of COVID-19 vaccination centers for these states were extracted from the vaccination center address lists officially published by the federal states.

To convert address information extracted from the official vaccination center address lists of the single states to geo-coordinates, all address data were subjected to an address geocoding routine using “Geokoder Bund” provided by the German Federal Office of Cartography and Geodesy prior to their integration in the COVID-19 vaccination center location dataset.

To be able to also consider the defined catchment areas of the COVID-19 vaccination centers in the accessibility model, as far as possible, all locational data of the COVID-19 vaccination centers as well as the analysis grid have been complemented by information on the catchment areas of the COVID-19 vaccination centers.

The available information suggests that differences exist between the federal states with regard to the areas of responsibility of the vaccination centers. In some federal states, vaccination centers are open to all residents independently from their distinct place of residence, whereas vaccination centers in other federal states only treat people assigned to a special vaccination center by place of residence. In Bavaria, for the assignment, the postal code of the place of residence is pivotal (see Fig. 1).

However, acquiring exact information on the detailed organization of the COVID-19 vaccination centers proved to be quite difficult. The reason is that although the responsible authorities in the federal states provide quite detailed information on the development and security of the vaccines, and the process of making an appointment in the vaccination centers (via the provided online portals) they only releases extremely sparse information on the detailed location of the vaccination centers or even the existence of the catchment areas of the single centers. So, the presented results on the accessibility of the COVID-19 vaccination centers are based on the available information on vaccination center locations as well as catchment areas at the time of the analysis (see Fig. 1).

### Accessibility Model

The accessibility of COVID-19 vaccination centers in Germany was analyzed with the help of an improved version of the Thünen-Accesssibility Model (Neumeier 2020). This model was developed for policy advice concentrating on geographic accessibility by determining travel cost (travel time) within traffic networks incurring for an individual to reach the next location of an infrastructure under consideration from the place of residence at the macro-level. As such, the model is primarily meant to answer the question whether a specific service or infrastructure is actually available or not within a certain commonly accepted travel time or distance. The advantage of this approach is that it can deliver a spatially detailed insight in the general accessibility situation as it presents itself for the households in Germany. However, the approach is not meant to take perceptions of accessibility into account, to analyze how accessibility presents itself for different groups of individuals or to take differences in service quality or options of choice into account.

**Fig. 2:**
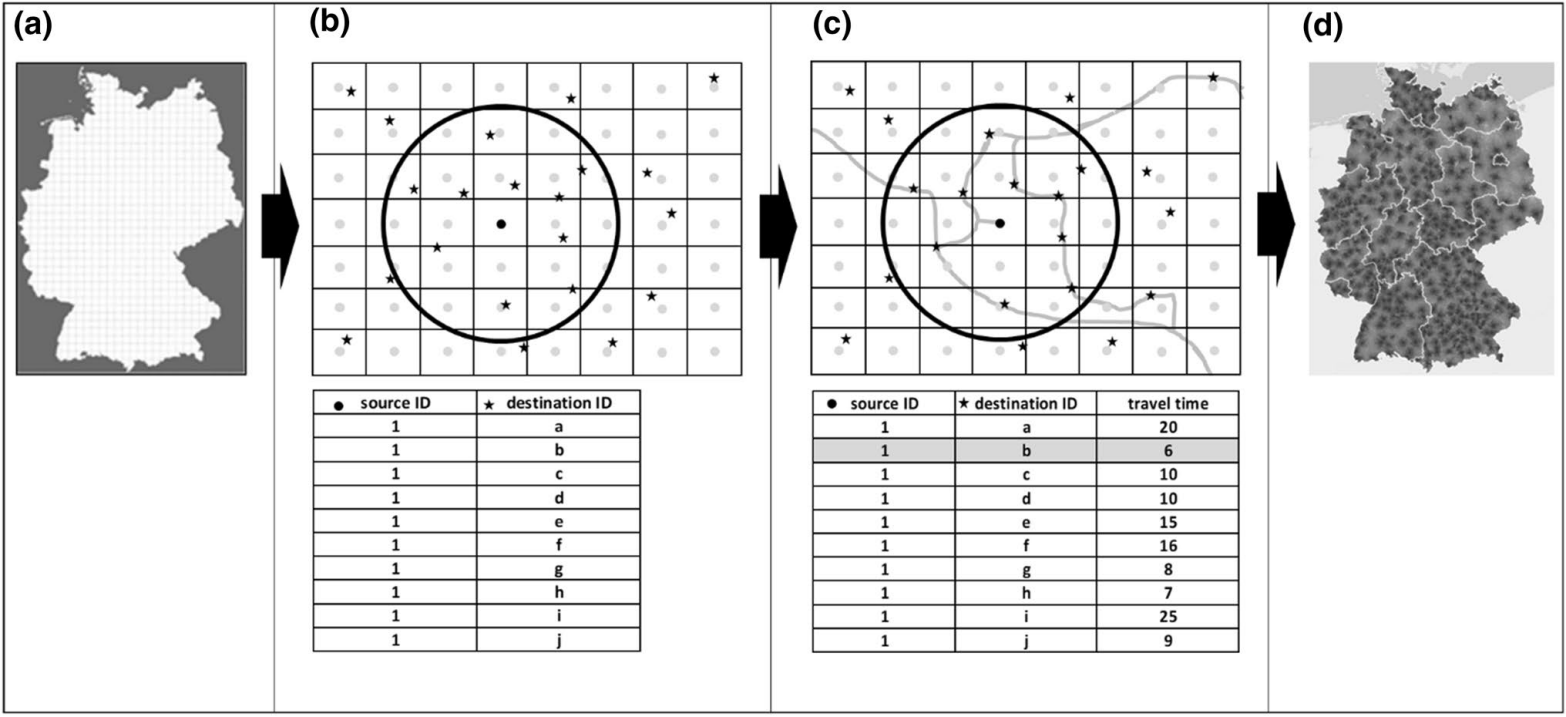
Conceptual design of the Thünen-Accessibility model. Illustration by author

To be able to obtain results below the level of the administrative unit of the communities and to be able to produce scalable results, it was decided to use a so-called “raster-based accessibility modeling approach” (Fig. 2). The peculiarity of this approach is, that the accessibility is not calculated for some kind of administrative units, or based on isochrones, but for the single cells of a small-scale vector-raster (grid) with which the area of interest—Germany, in our case—is overlain (Fig. 2a). That is, the centroids of the single grid cells represent the sources of the analysis, meaning that from every centroid, the shortest travel time within transport networks (streets/public transport networks) to the next COVID-19 vaccination center—within the defined catchment areas of the single centers (see Fig. 1)—is determined. The resulting travel time value is then attributed to the grid cell representing the travel time of this cell. Within-cell travel times are not considered (Fig. 2d). One prerequisite of this approach is, that the level of desired detail has to be balanced against computation costs. As the accessibility model is meant to also model accessibility by foot, another prerequisite is that the single grid cell is small enough so that the deviation of the walking distance from the centroid of the cell (that is used for the analysis) to the edges of the cell is not too large. Taking this aspect into consideration, presently a 250 m × 250 m grid covering the area of Germany was chosen as base.

As it turned out not to be possible to perform a many-to-many point analysis (from every grid cell centroid to every assigned COVID-19 vaccination center) for the whole German analysis grid with the hardware available, it was furthermore decided to base the calculation itself on a two-step process. First, for every centroid of the analysis grid, within the defined catchment areas of the vaccination centers, up to 10 nearest vaccination centers are determined by Euclidean distances.^12^ That is, if for a place of residence and its defined vaccination center catchment area, the possibility exists to choose between different possible vaccination centers, only up to the next 10 centers by Euclidean distance are considered in the analysis (Fig. 2b). Second, for every grid cell centroid of the analysis grid, the travel time to the travel time shortest COVID-19 vaccination center out of the ten possible centers defined by the euklidean data reduction process, is determined within the traffic network of the different means of transport analyzed (Fig. 2c).

Technically speaking, the accessibility model consists of two independent models. First, an accessibility model for the means of transport car, bicycle, foot (accessibility model street)—and principally other street-based means of transport—and second, an accessibility model for public transport, (accessibility model public transport) that is still in an experimental phase.^13^ The street network of Germany of the OpenStreetMap^14^ is used as traffic network for both models. In addition, for the accessibility model public transport, the street network is complemented with public transport time-table and route information of the whole German public transport system stored in the so-called General Transit Feed Specification (GTFS) format.

**Fig. 3:**
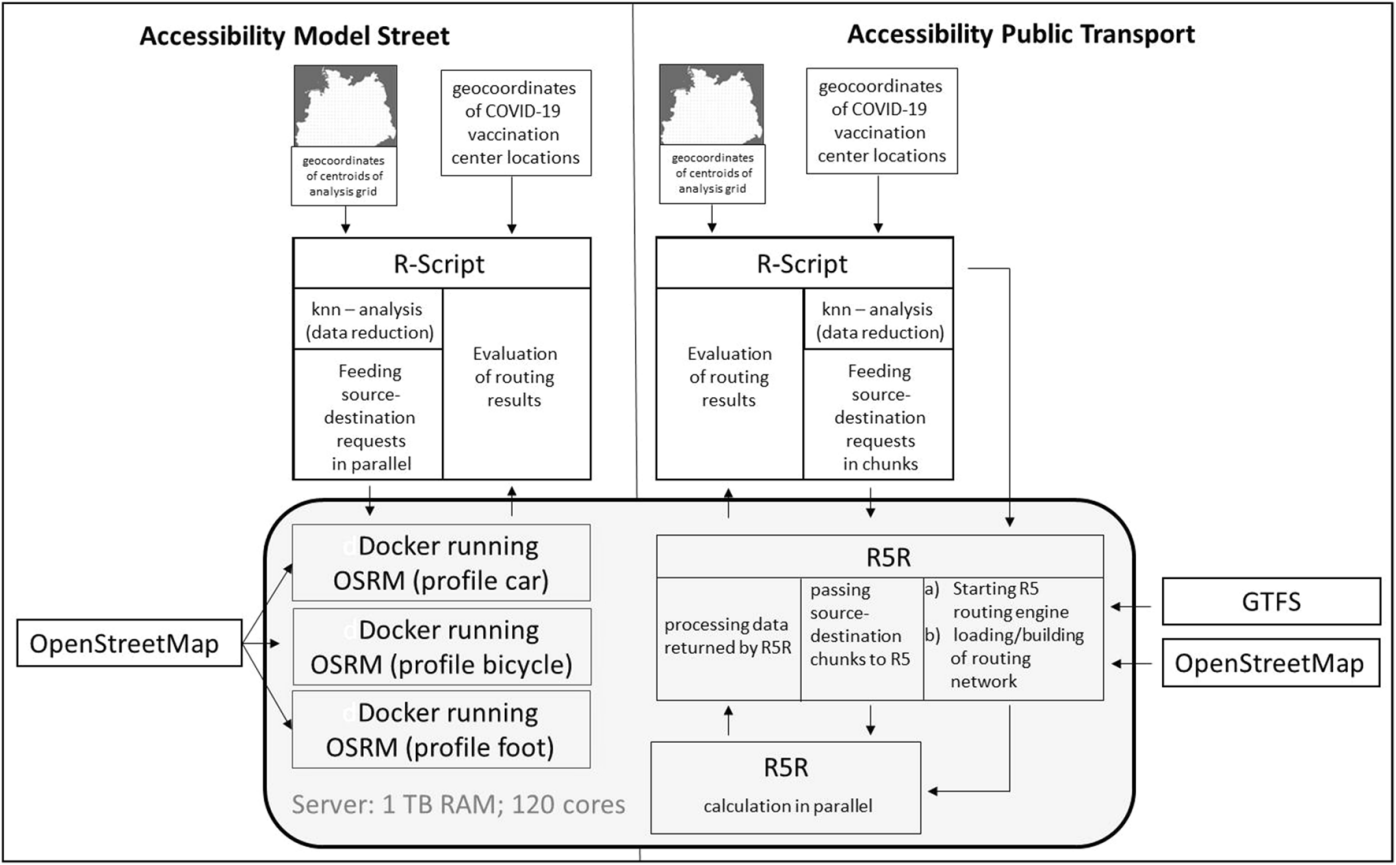
Conceptual design of technical realization of the accessibility models street and public transport. Illustration by author

Technically the accessibility model street is realized with the help of the Open Source Routing Machine (OSRM) version 5 via a “Multi Level Dijkstra Algorithm”.^15^ Calculated travel times are based on the speed profiles car, bicycle and foot as defined within the OSRM (Fig. 3).

The accessibility model public transport is realized with the help of R5R^16^ (Pereira et al. 2021) a R interface to the R5 routing engine developed by Conveyal^17^ (Fig. 3). Based on time table and route information, the R5R calculates travel times at specified start times from indicated sources to indicated destinations, thereby the walking times to and from public transport stops are as considered as initial waiting times and waiting times for necessary connections. Further, it was decided that the maximum walking distance of a single trip leg (distance to/from the public transport stops; distance between transfers) in the public transport analysis is restricted to 1.2 km (assuming a walking speed of 3.6 km/h, this corresponds approximately to a maximum walking time of 20 min per trip leg). The maximum allowed travel time was restricted to two hours. The maximum number of allowed transfers was limited to five. One peculiarity of the available public transport analysis frameworks based on R5 is the fact that they all have been developed for delivering information on the “best” public transport connections (e.g., lowest overall travel time) considering the provided date and time. As such, if the maximum allowed walking distance of the single trip legs chosen is too long the algorithms reach a situation where in a significant amount of cases “walking only” is the optimal solution (e.g., if by starting to walk right at the time entered as desired trip start the destination can be reached earlier than with first waiting for the means of transport and subsequently using the means of transport). On the other hand, if this value is set too low, possible feasible connections are omitted as the number of public transport stops that are considered as not reachable increase by decreasing the maximum allowed walking distance. So, on the one hand, to avoid an optimization for “walking only”, and on the other hand, to avoid the exclusion of too many reachable public transport stops, the maximum allowed walking distance per trip leg has to be chosen carefully. We decided to use a threshold of 1.2 km^18^ as this is a distance still feasible to walk to a public transport stop in rural areas and at the same time seems not to over-emphasize “walking only” connections. The two remaining parameters (maximum allowed travel time and maximum number of allowed transfers) directly influence the computation costs of the overall analysis. Again if set too low, the possible results are restricted and if set too high, the computation costs increase drastically. Considerations based on cost value as well as on the practicability for reaching COVID-19 vaccination centers led our decision in restricting the maximum allowed travel time as well as the maximum allowed transfers as described above.

## Accessibility of COVID-19 Vaccination Centers in Germany

Before we present the analysis results it has to be noted, that besides administering vaccinations in the COVID-19 vaccination centers, especially in the initial phase of the inoculation campaign, mobile teams called on nursing and retirement homes to administer vaccinations to the (mobility-reduced or hospitalized) residents on-site in addition. Furthermore, to facilitate the accessibility, some vaccination centers provide some kind of assisted transport services (sometimes restricted to the ways from/to the main train stations) for less mobile people. According to small amount of information released by the federal states on the organization of the inoculation campaign, there also seems to be additional vaccination centers established for certain times at certain places as temporary branches of existing centers temporarily complementing the existing centers if deemed necessary (e.g., on coastal islands and peninsulas). These are special cases mitigating accessibility restrictions for the least mobile people in affected regions that cannot be taken account of with our accessibility model that is meant for modeling accessibility at the macro-level of Germany. The main reason is that the data required to incorporate such region-specific peculiarities are not available countrywide. However, these aspects should be kept in mind when interpreting the results presented below.

**Fig. 4:**
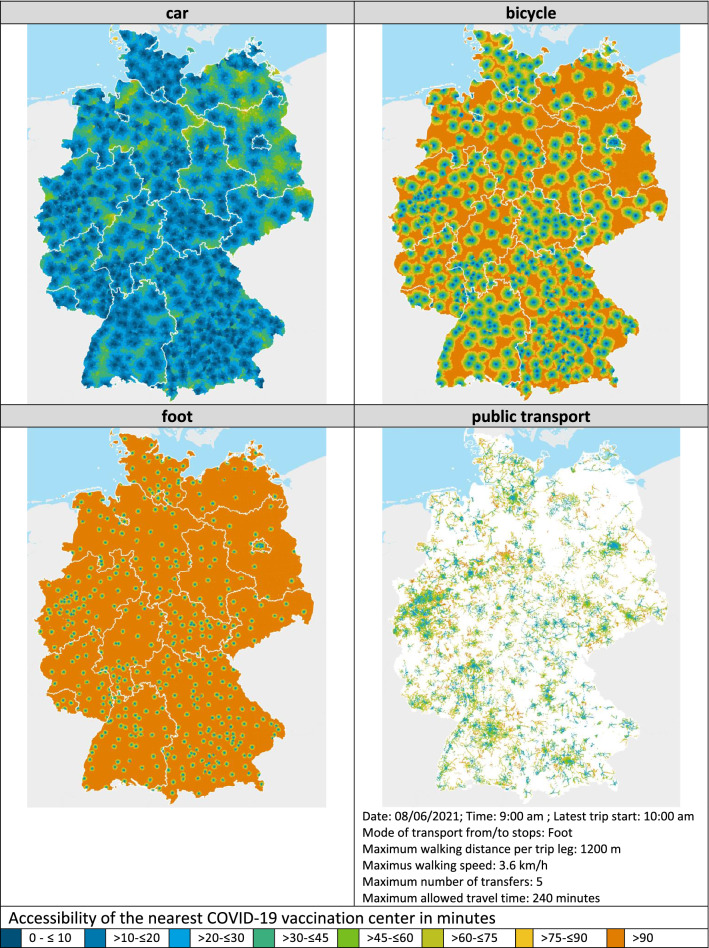
Accessibility of COVID-19 vaccination centers by different means of transport based on the calculated travel times for every cell of the 250 m × 250 m analysis grid. Administrative boundaries: German Federal Office of Cartography and Geodesy (2020); Data: Own calculations

The results of the accessibility analysis of COVID-19 vaccination centers in Germany for the different federal states and Thünen-types of rural regions are summarized in Table 2 in the annex of the paper. This table consists of two parts. In the first part of the table, the mean and median travel times by the considered means of transport car, bicycle and foot within the different Thünen-Types of rural regions are reported for Germany as well as the different federal states. Because public transport is not countrywide available at the chosen reverence time considering the public transport modeling parameters, it is not possible to report a mean and median public transport travel time valid for the whole region types listed. The second part of the table shows the percentage of the respective regions’ population that is able to reach the next COVID-19 vaccination center within the depicted travel time window. The color of the travel time windows in the table corresponds to the class colors depicted in the accessibility maps in Fig. 4 to enable a direct comparison.

### COVID-19 Vaccination Center Accessibility by Car

In Germany, all in all, the median travel time to the next COVID-19 vaccination center is approximately 22 min by car (see Table 2 in the annex). No great differences exist in the accessibility by car for people living in non-rural areas (17 min by car) and those living in rural areas (23 min by car). Concentrating on the rural region types only, in both the region types, “very rural regions with less good socioeconomic situation” and “rather rural regions with less good socioeconomic situation”, with 24 min to reach the next COVID-19 vaccination center, people living in this two region types experience the longest travel times by car out of all rural region types analyzed (see Table 2 in the annex).

Concentrating on the federal states, by car, the longest median travel times can be found in Brandenburg (33 min) followed by Mecklenburg-West Pomerania (30 min) and Saxony-Anhalt (26 min), the shortest in Bremen (13 min), followed by Berlin (16 min) and Schleswig–Holstein (18 min) (see Table 2 in the annex). Between the federal states, people living in rural areas have to accept median travel times between 18 min (Schleswig–Holstein) and  33 min (Brandenburg) by car (see Table 2 in the annex). In addition, Table 2 in the annex also shows that between the different federal states and the different rural region types (partly considerable), differences also exist as to the share of people that can reach a COVID-19 vaccination center within a defined travel time window.

### COVID-19 Vaccination Center Accessibility by Bicycle

In Germany, all in all, the median travel time to the next COVID-19 vaccination center is 76 min by bicycle (see Table 2 in the annex). By bicyle people living in non-rural areas can reach the next COVID-19 vaccination center in travel time of 51 min on median whereas people living in rural areas have to cycle 1 h and 19 min on median. As such, on median the rural population has to spend nearly half an hour more for accessing the next COVID-19 vaccination center by bicycle compared to that living in non-rural areas (see Table 2 in the annex). Concentrating on the rural region types only, it is the “very rural regions with less good socioeconomic situation” where with 1 h 24 min, the median travel time to the next COVID-19 vaccination center is longest for the analyzed means of transport bicycle (see Table 2 in the annex).

Concentrating on the federal states, by bicycle, the longest travel times can be found in Brandenburg with 1 h 53 min, the shortest in Bremen with 33 min. Between the federal states people living in rural areas have to accept median travel times between between 1 h (Schleswig–Holstein and Thuringia) and 1 h 54 min (Brandenburg) by bicycle Table 2 in the annex). In addition, Table 2 in the annex also shows that between the different federal states and the different rural region types (partly considerable), differences also exist as to the share of people that can reach a COVID-19 vaccination center within a defined travel time window.

### COVID-19 Vaccination Center Accessibility by Foot

In Germany, all in all, the median travel time to the next COVID-19 vaccination center is 3 h 24 min by foot (see Table 2 in the annex). On foot, the median travel time in non-rural areas is with 2 h 8 min considerably lower than in rural areas with 3 h 32 min (see Table 2 in the annex). However, the median travel times by foot clearly show that for greater parts of non-rural and rural areas alike the mode of transport foot is not a feasible option to access a COVID-19 vaccination center.

Concentrating on the rural region types only,it is again the “very rural regions with less good socioeconomic situation” where the median travel time with 3 h 48 min to the next COVID-19 vaccination center is longest for the analyzed means of transport foot (see Table 2 in the annex). Concentrating on the federal states, by foot, the longest travel times can be found in Brandenburg with 5 h 5 min, the shortest in Bremen with 1 h 21 min (see Table 2 in the annex). Between the federal states, people living in rural areas have to accept median travel times between between between 2 h 35 min (Thuringia) and 5 h, 6 min (Brandenburg) by foot (see Table 2 in the annex). In addition, Table 2 in the annex also shows that between the different federal states and the different rural region types (partly considerable), differences also exist as to the share of people that can reach a COVID-19 vaccination center within a defined travel time window.

### COVID-19 Vaccination Center Accessibility by Public Transport

Considering the use of public transport, it is not possible to report an overall median travel time as with the other analyzed means of transport. The reason is that at the reference time (June 8, 2021; departure between 9 to 10 am) of the analysis only approximately 78% of the people can make use of public transport whereas for 22% no public transport opportunity exists.^19^ However, for the 78% of the population that can use public transport the median travel time is 53 min. Interestingly, in non-rural regions 96% of the people can use public transport to access the next COVID-19 vaccination center. These 96% need a median travel time of 47 min. In contrast, only 65% of the people living in rural areas can use the public transport to access the next COVID-19 vaccination center. These 65% need a median travel time of 58 min (see Table 2 in the annex). The lowest share of people who can make use of public transport can be found in “very rural regions with less good socio-economic situation” (share of people 54%, median travel time 54 min), followed by “very rural regions with good socioeconomic situation” (share of people 58%; median travel time 56 min), “rather rural regions with less good socio-economic situation” (share of people 71%; median travel time 58 min) and “rather rural regions with good socio-economic situation” (share of people 75%; median travel time 56 min).

In the federal states, the share of people that can use public transport to access the next COVID-19 vaccination center is between 50% (Mecklenburg-West Pomerania) and 100% Bremen (see Table 1). Considering only the rural areas in the federal states, the share of people that can use public transport is between 44% (Mecklenburg-West Pomerania) and 88% (Saarland) (see Table 1). In rural areas, people living in Saxony will experience the longest median travel times using public transport (65 min) to reach the next COVID-19 vaccination center. The shortest median public transport travel times can be found in rural areas in Thuringia with 47 min (see Table 1).

Interestingly, for the people that can use public transport in rural regions due to the accessibility model, in no federal state does the median public transport travel times to the next COVID-19 vaccination center exceed 65 min. However, due to the accessibility model, considering the different rural region types separately differences exist, such as in the share of people that can make use of public transport as in the median travel times (see Table 1). In rural Germany, according to the analysis, “very rural regions with less good socio-economic situation” in Bavaria show the lowest share of people that can use public transport to access the next COVID-19 vaccination center (see Table 1). Among these regions Saarland has the highest share of people that can make use of public transport. In “rather rural regions with less good socioeconomic situation” in Saarland 97% of the people can use a bus or train (see Table 1). From the people that can use public transport according to the accessibility model, people living in “rather rural regions with good socio-economic situation” in Saarland experience, with 1 h 17 min, the longest public transport travel times. People living in Thuringia in “rather rural regions with less good socio-economic situation” have the shortest routes with 44 min (see Table 1). Again, in addition, Table 2 in the annex also shows that between the different federal states and the different rural region types (partly considerable) differences also exist as to the share of people that can reach a COVID-19 vaccination center within a defined travel time window.

For example, in Saarland, in the “rather rural regions with good socioeconomic situation”, 76% of the people will experience public transport travel times greater than 60 min and only 11% of the people can expect shorter public transport travel times (see Table 2 in the annex).

**Table 1:**
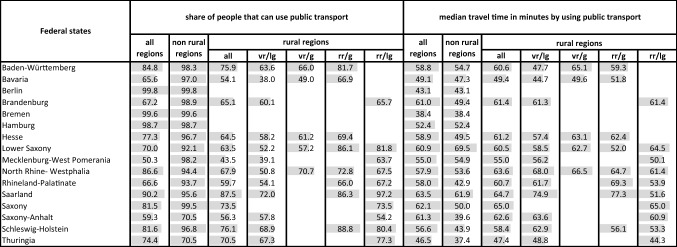
Share of people that can use public transport to access the next COVID-19 vaccination center in the federal states, types of rural areas and median travel times using public transport

vr/lg: very rural, less good socio-economic situation

br/g: very rural, good socio-economic situation

rr/g: rather rural, good socio-economic situation

rr/lg: rather rural, less good socio-economic situation

Calculations by author

**Table 2:**
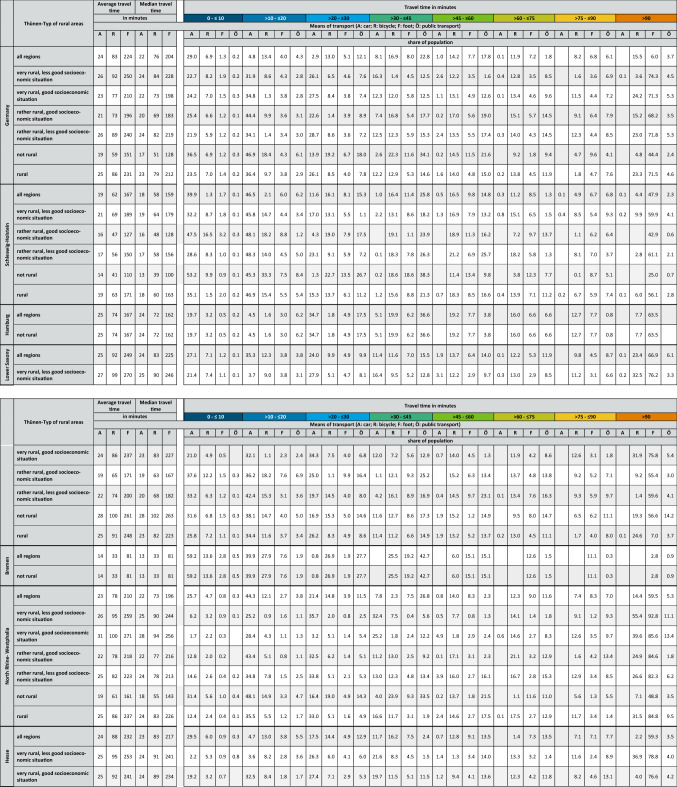

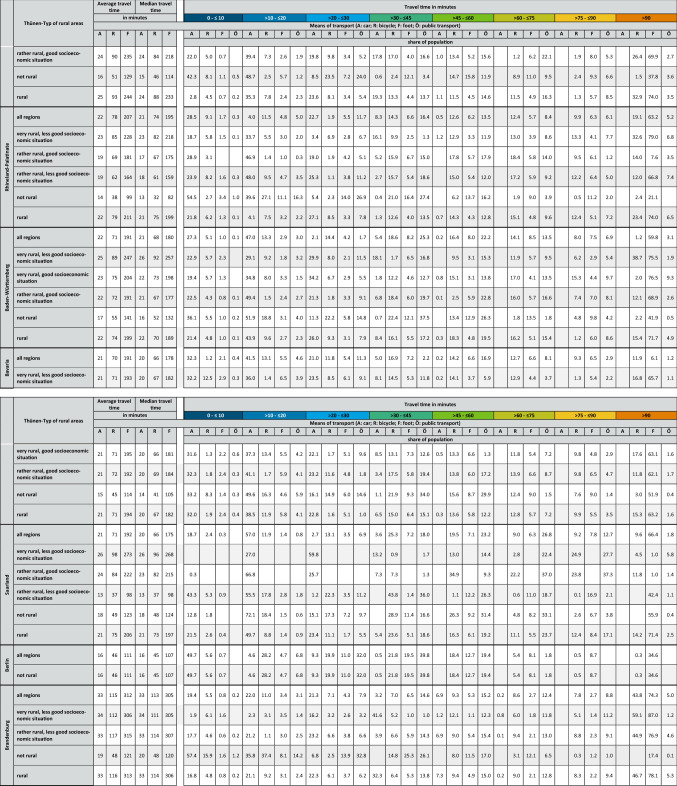

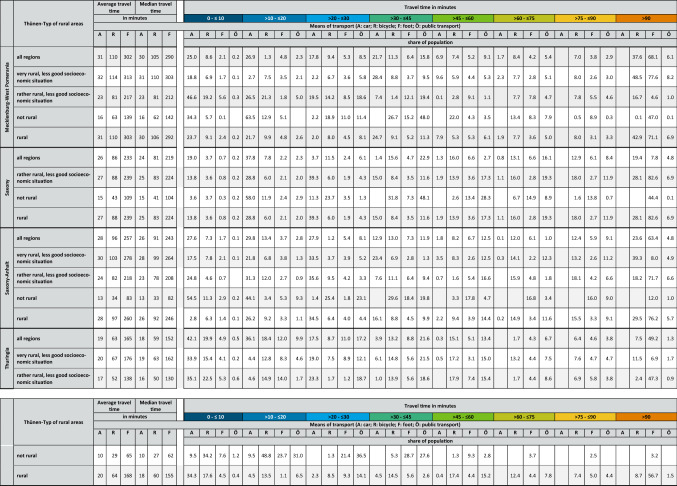
Accessibility of COVID-19 vaccination centers by means of transport, types of Rural Regions, federal states and population

Calculation by author

### Synthesis: Spatial Accessibility Patterns by Means of Transport

**Fig. 5:**
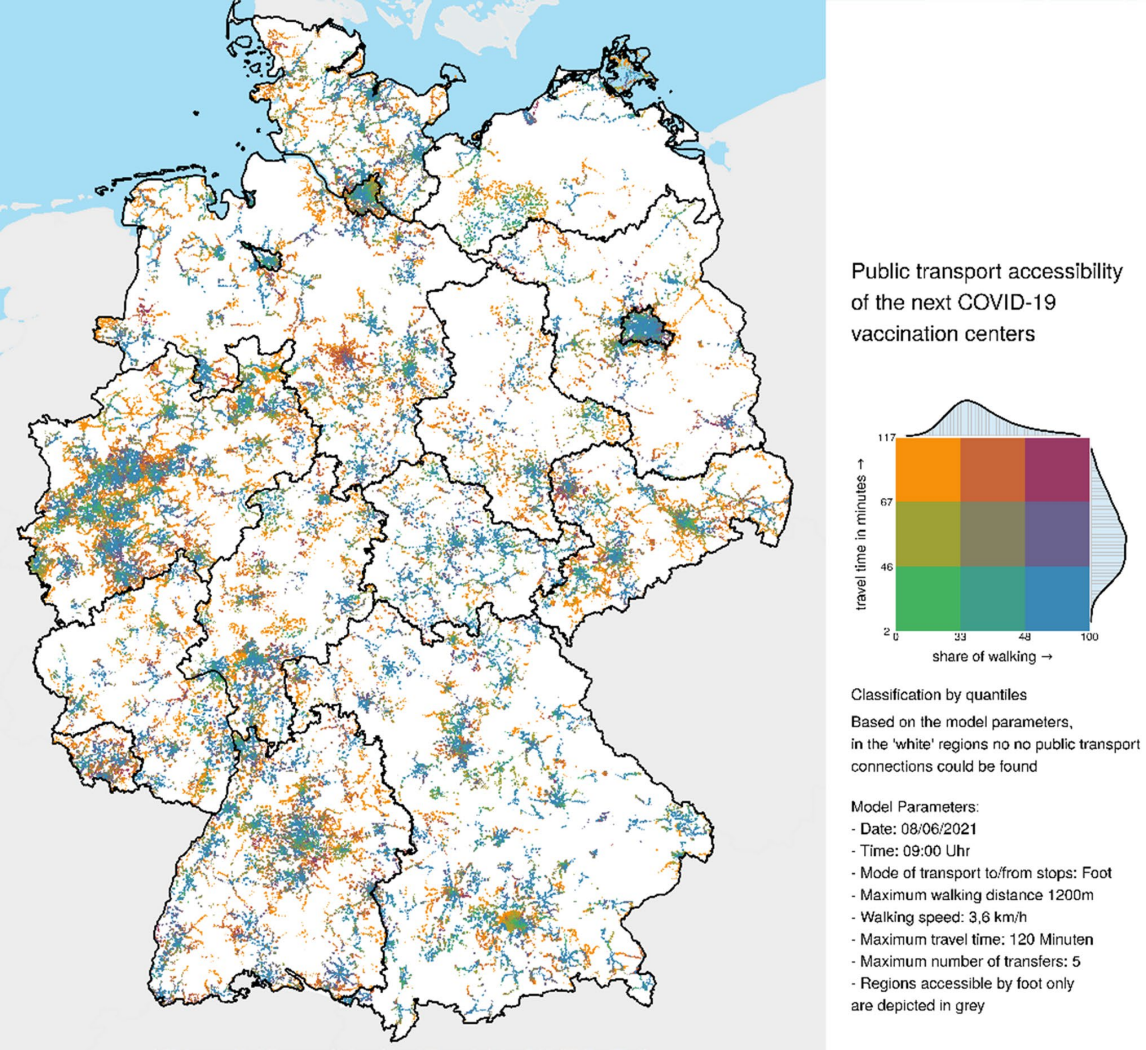
Public transport travel time to the next COVID-19 vaccination center and share of walking in travel time. Administrative boundaries: German Federal Office of Cartography and Geodesy (2020); Data: Own calculations

How this differentiated accessibility situations using the different means of transport car, bicycle, foot and public transport characterized above present itself in a spatial sense is presented in Figs. 4 and 5.

The four maps in Fig. 4 show the accessibility situation of COVID-19 vaccination centers differentiated by the analyzed means of transport car, foot, bicycle and public transport.^20^The map in Fig. 5 allows for the multimodal character of public transport travel by showing the public transport travel time to the next COVID-19 vaccination center together with the share of walking in total travel time. The above-described picture can also be identified here. The maps clearly show that by car COVID-19 vaccination centers are relatively well accessible. Nevertheless, especially in Brandenburg and Mecklenburg-West Pomerania, there are regions with comparably long travel times (more than 30 min). And also in the other federal states such regions can be identified, but to a lesser extent (green to yellow colors). However only roughly 9% of the German population lives in these regions (see Table 2 in the annex). The maps for bicycle and foot draw a slightly different picture that clearly shows that virtually only in the settlement centers and its nearer surroundings these means of transport are a theoretical option. In 30 min by bicycle, 67% of the German population cannot reach a COVID vaccination center, on foot, this is the case for 90% of the German population (see Table 2 in the annex). The public transport map shows that the farther away people live from the main settlement centers the longer the travel times that have to be accepted are (Fig. 4). In addition, Fig. 5 shows that using public transport, the share of walking in total travel time is higher in the non-rural regions and main rural settlement centers and decreases in the outskirts of the non-rural regions and main rural settlement centers. In contrast when using public transport, the total travel times are in the range of the lower tertile (up to 46 min) in the non-rural regions and main rural settlement regions, whereas outside these regions, they are in the range of the upper tertile (from 67 min onwards) (Fig. 5). This peculiarity, the higher share of walking in non-rural regions and the rural settlement centers, might in part explain the slightly longer observed overall travel time when using public transport in these areas, for the other part, it can be guessed that the augmented stop frequency and reduced driving speed street are a crucial factor.

Another aspect that might especially in urban areas influence the accessibility on foot as by public transport alike is the specific requirement on the built infrastructure to be suitable as possible vaccination center location. That is, to provide ample space for the inoculation campaign and the expected rash of visitors. As such, especially in urban areas, fair halls or industrial buildings offside from residential areas with reduced frequency of use by public transport have been chosen as vaccination center locations.

## Discussion and Conclusion

Considering the aspects presented above, all in all, with regard to the accessibility of COVID-19 vaccination centers in Germany, the results of the accessibility analysis are unambiguous: for mobile people that can use a car, the accessibility of the next COVID-19 vaccination center should not present any problems regardless if they live in non-rural or rural areas. The same applies for the majority of people living in urban and rural areas that can make use of public transportation. Considering Germany as a whole, only 20% of the population will experience travel times longer than 60 min when using public transportation, however, a differentiated analysis of the public transportation accessibility within the different rural region types and the different federal states reveals that there are also disadvantaged regions when it comes to using public transportation, especially in Saarland. However, with a few exceptions, the share of people that can use public transport to reach the next COVID-19 vaccination center in a still feasible overall travel time is thankfully rather high. However, especially persons who have difficulty walking might especially in rural areas experience challenges to cover the distance to/from the public transport stops. With limitations, for people living in the settlement centers or their nearer surroundings, using a bicycle is in principle an option too. However, it can be supposed that especially for the most vulnerable or elderly people, this means of transport is not a viable option. Regarding access on foot, a population share of only 10% (including non-rural regions) can reach the next COVID-19 vaccination center in less than 30 min. So, if one does not live in close proximity to the next COVID-19 vaccination center, the two means of transport, bicycle and foot, seem to be of secondary importance for most people and especially the rural population.

In summary, the observations on the COVID-19 vaccination center accessibility together with this consideration suggest that people leading a self-reliant life in their own residence, but with restricted mobility who cannot use a car or transport service might most likely experience problems in accessing COVID-19 vaccination centers. It can be supposed that these people most likely belong to the group of people most vulnerable susceptible for a difficult etiopathology.

All in all, from the macro-level point of view of the German-wide accessibility analysis, there is strong evidence that living in the same (rural) neighborhood, the question about adequate accessibility depends on the individual mobility and varies between those who can use a car and those who cannot. Hence, COVID-19 vaccination center accessibility is likely to be more of an individual experience, depending more on individual living conditions and capabilities than on a spatially distinct phenomenon cheating non-rural regions and discriminating rural regions as initially anticipated. As such one can conclude that the state governments succeeded in the location decisions and establishment of the COVID-19 vaccination centers. They managed to establish an efficient local network that is in principle able to provide adequate access countrywide for COVID-19 vaccinations for the majority of the German population.

However, in practice, there are also some flaws, supporting the impression of poor accessibility in some places or states, but cannot be taken into consideration with an accessibility model. The availability of ample vaccines exempted, in some federal states appointments will not necessarily always be possible at the next (by distance or travel time) vaccination center, but in the next vaccination center with appointments available everywhere within the defined catchment areas. These can in some federal states be practically everywhere within the state and as such for the people affected could indeed be individually difficult to access from the place of residence. Another aspect is whether adequate assistance exists for people with restricted mobility to get to the COVID-19 vaccination center at the date fixed. So, to fully evaluate the accessibility of the COVID-19 vaccination centers (the aspect of the availability of ample vaccines excluded) our analysis results on access by different means of transport should be supplemented by future research on individual experiences in practice. In this context, we also think it would be interesting to not only concentrate on accessibility by transport, but on the overall accessibility of getting an appointment with special attention on experiences of less technophile people too.

Another interesting research question for further analyses taking up the findings presented in this paper could be the reflection of the vaccination rate with the accessibility of the vaccination centers in order consider whether and if so how vaccination center accessibility influenced the utilization of the COVID-19 vaccination.

Together with our findings on the accessibility of COVID-19 vaccination centers, such findings can help to uncover and understand the potential shortcomings of the COVID-19 inoculation campaign in preparation of an even more targeted reaction to potential future pandemic outbreaks or other situations that require reaching great parts of the population in an efficient manner.

However, one interim result based on our findings on traffic accessibility of COVID-19 vaccination centers is, that should a similar campaign be necessary in a future disaster response, additional measures seem to be necessary to especially support less mobile people leading a self-reliant life in accessing the service centers regardless from their place of residence. Another aspect for improvement suggested by our findings could be an enhancement of public transportation, respectively, the establishment of a system to support people in accessing frequently served public transport stops, in areas still underserved (as a result of the rail closures observable especially in rural areas in the last years).

## Data Availability

Not applicable.

## Appendix

See Table 2.

## Footnotes

1 Epidemische Lage von Nationaler Tragweite. Bis zum 28.3.2020 gab es in Deutschland 48.582 bestätigte Fälle und 325 Todesfälle (Robert Koch Institut 2020b).

2 See for example:

- https://www.waz.de/staedte/muelheim/impfzentrum-muelheim-kritik-an-umstaendlicher-erreichbarkeit-id231592521.html (07.06.2021).
- https://www.neuepresse.de/Hannover/Meine-Stadt/Impfzentrum-Senioren-scheuen-die-lange-Anfahrt (07.06.2021).
- https://www.wochenspiegellive.de/eifel/kreis-bitburg-pruem/pruem/artikel/teurer-weg-zum-impfen-wer-weit-weg-wohnt-hat-pech-69093/ (07.06.2021).
- https://www1.wdr.de/nachrichten/alternativen-zu-schwer-erreichbaren-impfzentren-100.html (13.07.2021).
- https://n-land.de/lokales/nuernberger-land/schwieriger-zugang-zur-corona-schutzimpfung (13.07.2021).
- https://www.mdr.de/nachrichten/sachsen/chemnitz/vogtland/streit-bahn-verkehr-haltepunkt-eich-100.html (13.07.2021).
- https://www.svz.de/lokales/prignitz/Anreise-zum-Impfzentrum-Ein-langer-Weg-fuer-die-Spritze-id30823402.html (13.07.2021).
- https://www.freiepresse.de/vogtland/plauen/corona-lage-im-vogtland-die-lange-anreise-zum-impfzentrum-artikel11315529 (13.07.2021).

3 In this context measures are addressed that take a gradual decrease in accessibility by increasing travel costs into consideration (Handy and Niemeier 1997).

4 Opportunity indicators measure the ease with which activity opportunities can be reached from a given location. They can be measured using the cumulative amount of opportunities from an origin within a given amount of travel time (Chen et al. 2011).

5 Utility based measures approach accessibility on an individual level (Handy and Niemeier 1997).

6 In this context measures are addressed that measure interactions between all the possible location pairs (Rodrigue 2020).

7 Potential models measure interactions between one location and every other location (Rodrigue 2020).

8 Retail models measure the boundary of the market areas between locations (Rodrigue 2020).

9 Population data are obtained from the gridded population statistics (100 m × 100 m grid) provided by the German Federal Office for Statistics, based on the 2011 census projected into the analysis grid. At the time of conducting the analysis this ten year old gridded population dataset is the most recent official small-scale population data available for Germany. As such the population values reported in the analysis should be interpreted as “rough housenumbers” only.

10 The regional differentiation in the analysis follows the Thünen-Typology of Rural Areas. This is a classification based on several structural as well as socio-economic indicators developed to explicitly differentiate different kinds of rural areas based on their structural performance in Germany. This typology considers rural regions as regions with a low settlement density and population in the surroundings of the regions, low-density areas and a relatively high share in agricultural and forestry areas that are located in a peripheral position to the economic and population centers (Küpper 2016). These rural areas are then further differentiated in regions with good and less good socio-economic performance (Küpper 2016).

11 Sources, see section information on vaccination center locations and catchment areas in the reference section.

12 It was decided to take the ten next possible locations into consideration as experiences showed that by focusing on 10 locations possible allocation errors are minimized by still acceptable computation times.

13 The reason is, that modeling public transport accessibilities is much more complex and computationally expensive that modeling accessibilities in street networks.

14 https://www.openstreetmap.org (08.06.2021).

15 http://project-osrm.org (08.06.2021).

16 https://github.com/ipeaGIT/r5r(08.06.2021).

17 https://github.com/conveyal/r5(08.06.2021).

18 We are aware that for people who have difficulty walking or people with other mobility constraints, traveling 1.2 km (per trip leg) by foot can present an insuperable barrier, but are however especially in rural areas longer distances to public transport stops the reality.

19 These include people living in regions with no scheduled public transport opportunity at the given reference time as well as people that could in principle make use of public transport, but would need more than five transfers, travel more than four hours or would have to walk more than 1.2 km per trip leg. That is, situations which we equalized with “not accessible by public transport” for the reasons explained in the methodology section.

20 In order to enable the visual detection of global spatial accessibility patterns it was decided to map in each case the entire 250 m × 250 m analysis analysis grid and not only the populated grid cells of the analysis grid.
